# ELK4 promotes the development of gastric cancer by inducing M2 polarization of macrophages through regulation of the KDM5A-PJA2-KSR1 axis

**DOI:** 10.1186/s12967-021-02915-1

**Published:** 2021-08-09

**Authors:** Lei Zheng, Hongmei Xu, Ya Di, Lanlan Chen, Jiao Liu, Liying Kang, Liming Gao

**Affiliations:** 1 Department of Oncology, The First Hospital of Qinhuangdao, No. 258, Wenhua Road, Qinhuangdao, 066000 Hebei Province People’s Republic of China; 2 Department of Oncology, Tianjin Wuqing District People’s Hospital, Tianjin, 301700 People’s Republic of China

**Keywords:** Gastric cancer, Macrophages, Transcription, Methylation, Ubiquitination, ELK4, KDM5A, PJA2, KSR1

## Abstract

**Background:**

We tried to elaborate the molecular mechanism of ETS-like transcription factor 4 (ELK4) affecting gastric cancer (GC) progression through M2 polarization of macrophages mediated by lysine-specific demethylase 5A (KDM5A)-Praja2 (PJA2)-kinase suppressor of ras 1 (KSR1) axis.

**Methods:**

GC expression dataset was obtained from GEO database, and the downstream regulatory mechanism of ELK4 was predicted. Tumor-associated macrophages (TAMs) were isolated from GC tissues. The interaction among ELK4, KDM5A, PJA2 and KSR1 was analyzed by dual luciferase reporter gene, ChIP and Co-IP assays. The stability of KSR1 protein was detected by cycloheximide (CHX) treatment. After TAMs were co-cultured with HGC-27 cells, HGC-27 cell biological processes were assessed through gain- and loss-of function assays. Tumorigenicity was detected by tumorigenicity test in nude mice.

**Results:**

In GC and TAMs, ELK4, KDM5A and KSR1 were highly expressed, while PJA2 was lowly expressed. M2 polarization of macrophages promoted the development of GC. ELK4 activated KDM5A by transcription and promoted macrophage M2 polarization. KDM5A inhibited the expression of PJA2 by removing H3K4me3 of PJA2 promoter, which promoted M2 polarization of macrophages. PJA2 reduced KSR1 by ubiquitination. ELK4 promoted the proliferative, migrative and invasive potentials of GC cells as well as the growth of GC xenografts by regulating KSR1.

**Conclusion:**

ELK4 may reduce the PJA2-dependent inhibition of KSR1 by transcriptional activation of KDM5A to promote M2 polarization of macrophages, thus promoting the development of GC.

**Supplementary Information:**

The online version contains supplementary material available at 10.1186/s12967-021-02915-1.

## Background

Gastric cancer (GC) is regarded as the fifth prevalent malignancy and the third leading reason for cancer-related deaths on a global scale [1]. The risk factors for the occurrence of GC include geographical location, dietary habit, in addition to genetic background of the host [2]. Gastrectomy combined with systematic lymph node dissection can be curative for GC but leads to postoperative complications [3]. Chemotherapy is a recommended treatment regimen for patients with advanced GC, but the chemotherapy resistance still poses an obstacle to the efficacy [4]. Importantly, tumor-associated macrophages (TAMs), exerting a pivotal role in the tumor microenvironment, can be categorized into M1 and M2 phenotypes and engaged GC progression [5].

ETS-like transcription factor 4 (ELK4) is a member of the ternary complex factor subfamily of E twenty-six domain transcription genes [6]. Intriguingly, ELK4 can regulate cellular homeostasis and stress responses in macrophages to affect acute responses to external infection [7]. Lysine-specific demethylase 5A (KDM5A/RBP2) is identified as a chromatin-modifying enzyme related to transcriptional regulation by catalyzing removal of methyl groups from methylated lysine 4 of histone H3 and this gene is observed in multiple cancers including GC [8]. As previously reported, suppression of KDM5A by microRNA-212 could result in inhibited GC proliferation [9]. Praja2 (PJA2), belonging to the growing family of mammalian RING E3 ubiquitin ligases, has been found to be implicated in different cancers and neurological diseases [10]. A previous study suggests that PJA2 promotes the accumulation of ubiquitinated MFHAS1 but does not degrade it; MFHAS1 ubiquitination by PJA2 positively regulates the TLR2-mediated JNK/p38 pathway and promotes M1 macrophage polarization, M2 to M1 macrophage transformation and inflammatory response [11]. Another study has shown that low expression of GADD45A, PPP1CB, PJA2, and KLF2 is associated with poor overall survival [12], which provides insight into the underlying mechanisms of GC pathogenesis. Downregulation of PJA2 was revealed to share association with worse overall survival of patients with GC [12]. Strikingly, PJA2 has been identified as the E3 ligase which can ubiquitylate kinase suppressor of ras 1 (KSR1) [13], a molecular scaffold of the Raf/MEK/extracellular signal-regulated kinase (ERK) cascade which can augment oncogenic Ras signaling [14]. Of note, KSR1 has been identified as a possible biomarker to predict the response for RAD001 containing treatment in patients with advanced GC [15]. Considering all the above findings, we propose a hypothesis in the current study that ELK4 may affect the development of GC by regulating macrophages, with the involvement of the KDM5A-PJA2-KSR1 axis.

## Materials and methods

### Ethical approval

The study was approved by the ethics committee of the First Hospital of Qinhuangdao. This study was in line with the *Declaration of Helsinki*, and all patients signed informed consent.

### Bioinformatics analysis

Through the GEO database (https://www.ncbi.nlm.nih.gov/geo/), a GC expression microarray GSE29998 was obtained, which included 49 normal samples and 50 tumor samples. Differential analysis was performed using the R language “limma” package. Differential p-values were corrected using the FDR method and GC-related significantly differentially expressed genes were obtained with |logFC| > 1, adj.p.val < 0.05 considered as differential gene screening criteria. Through the cistrome database (http://cistrome.org/), human transcription factor data were obtained, and the list of 318 Translate factors (TFs) were downloaded [16]. Using the GeneMANIA database (http://genemania.org/), candidate transcription factors were subjected to correlation analysis, and correlation scores were obtained. GeneMANIA (www.genemania.org), an online analysis tool for deriving hypotheses based on gene functions, can search and create a list of genes with same functions to the target gene and show the correlation between the target gene and the data set by plotting an interactive network [17]. GEPIA2 is based on TCGA (https://tcga-data.nci.nih.gov/tcga/) and the GTEx (https://www.gtexportal.org/home/index.html) databases with a total of 84 cancer subtypes analysis. Through the GEPIA2 database (http://gepia2.cancer-pku.cn/#index), differential expression of the candidate genes in TCGA and GTExGC databases and differentially expressed genes in GC were obtained. Downstream genes of ELK4 were gathered using the ChipBase database (http://rna.sysu.edu.cn/chipbase/index.php). ChIPBase is a database for decoding the transcriptional regulation of mRNAs [18]. The LinkedOmics (http://www.linkedomics.org/login.php/) is an online analysis platform that applies multi-omics data of 32 TCGA cancers types for multi-dimensional analysis [19]. Besides, we identified overlapped genes associated with ELK4 in GC through the “LinkedCompare” module in linkedOmics, and the results were shown by Venn plot. Through the STRING database (https://string-db.org), a database of known and predicted protein–protein interactions [20], correlation analysis of ELK4 downstream candidate genes was performed to construct gene interaction network maps by cytoscapev3.7.1 software, and core gene degree values were counted.

### Clinical samples

GC tissues and adjacent tissues were collected from 30 patients with GC hospitalized in the First Hospital of Qinhuangdao from May 2016–May 2020, including 15 males and 15 females, aged from 28 to 68 years, with a median of 50 years. Adjacent tissues were non-tumor tissues no more than 5 cm beside the GC tissues. None of the GC patients in this study received chemotherapy, radiotherapy or immunotherapy before surgery.

### Isolation and culture of primary TAMs

Fresh GC tissues were cut into small pieces and digested in collagenase B (1 mg/ml, 11088807001, Roche Diagnostics GmbH, Mannheim, Germany) containing buffer A. The dissociated cells were collected into 15 ml test tubes and centrifuged at 400*g* for 5 min. TAMs were isolated from the sediment using Percoll density gradient centrifugation Kit (17-0891-01, Pharmacia) according to the manufacturer’s instructions.

### Co-culture of M2/M1 macrophages and GC cells

THP-1 macrophages were treated with 100 ng/ml PMA Sigma-Aldrich Chemical Company, St Louis, MO, USA) for 30 h to produce THP-1 macrophages (M0 macrophages), and then induced with IL-4 (50 ng/ml, R&D Systems, Minneapolis, MN, USA) for 48 h to polarize into M2 macrophages. They were continuously induced with LPS (100 ng/ml, Sigma) for 48 h to polarize into M1 macrophages. M2/M1 macrophages were co-cultured with GC cells HGC-27 (Product Number: ZQ0192, Zhongqiao Xinzhou Biotechnology Co., Ltd., Shanghai, China) in a 6-well cross well co-culture system (well size: 0.4 μm, Corning Glass Works, Corning, NY., USA). After 48 h, the co-cultured GC cells were collected for subsequent experiments.

### Cell transfection and grouping

TAMs were divided into: sh-NC (transfection of negative control plasmid of gene silencing), sh-ELK4 (transfection of silencing plasmid of ELK4), sh-NC + oe-NC (transfection of negative control plasmids of gene silencing and overexpression), sh-ELK4 + oe-NC (transfection of ELK4 silencing plasmid and gene overexpression negative control plasmid), sh-ELK4 + oe-KDM5A (transfection of ELK4 silencing plasmid and KDM5A overexpression plasmid), sh-KDM5A + sh-NC (transfection of KDM5A silencing plasmid and gene silencing negative control plasmid), sh-KDM5A + sh-PJA2 (transfection of KDM5A and PJA2 silencing plasmids), oe-NC (transfection of KDM5A gene overexpression negative control plasmid), oe-PJA2 + oe-KSR1 (transfection of PJA2 and KSR1 overexpression plasmid), and sh-ELK4 + oe-KSR1 (transfection of ELK4 silencing plasmid and KSR1 overexpression plasmid) groups. Lipofectamine 2000 (Invitrogen, Carlsbad, CA, USA) kit (11,668,019, purchased from Thermo Fisher) was used for transfection. After 48 h of culture, the cells were collected to detect the transfection effect for subsequent experiments.

### Reverse transcription-quantitative polymerase chain reaction (RT-qPCR)

Trizol (Invitrogen, Carlsbad, CA, USA) was used to extract total RNA from tissues and cells. Nanodrop2000 micro ultraviolet spectrophotometer (1011U, NanoDrop Technologies Inc., Wilmington, USA) was used to detect the concentration and purity of total RNA. According to the instructions of TaqMan MicroRNA Assays Reverse Transcription primer (4427975, Applied Biosystems, Carlsbad, CA, USA), the total RNA was reversely transcribed into cDNA. The primers of ELK4, KDM5A, PJA2, TNF, NOS2, Fizz1, Ym1, and Arg-1 were designed and synthesized by Takara company (Dalian, China) (Additional file 1: Table S1). Real time fluorescent qPCR was performed by ABI7500 qPCR instrument (7500, ABI, Oyster Bay, NY, USA). Using glyceraldehyde-3-phosphate dehydrogenase (GAPDH) as internal reference, the relative transcription levels of target genes were calculated by relative quantitative method (2^−∆∆Ct^ method).

### Western blot analysis

Total protein was extracted from tissue or cell by radioimmunoprecipitation assay (RIPA) lysate containing PMSF (P0013C, Beyotime, Shanghai, China) followed by protein concentration detection. The samples were then transferred to a polyvinylidene fluoride membrane after sodium dodecyl sulfate polyacrylamide gel electrophoresis (SDS-PAGE). After that, the membrane was blocked with 5% skim milk powder at room temperature for 1 h and incubated overnight at 4 °C with diluted primary rabbit antibodies against ELK4 (1:1000, ab86002, Abcam, Cambridge, MA, USA), KDM5A (1:5000, ab194286, Abcam), PJA2 (1:800, ab131118, Abcam), KSR1 (1:1200, ab68483, Abcam), and GAPDH (ab9485, Abcam; internal reference). The membrane was then incubated with HRP-labeled goat anti-rabbit against IgG H&L (ab97051, 1:2000, Abcam) for 1 h. Subsequently, the membrane was developed by the enhanced chemiluminescence kit (BB-3501, Ameshame, UK). The images were taken by Bio-Rad image analysis system (Bio-Rad Laboratories, Hercules, CA, USA) and analyzed by Quantity One v4.6.2 software.

### Enzyme-linked immunosorbent assay (ELISA)

GC tissues and adjacent tissues were homogenized with precooled PBS and centrifuged at 800*g* for 10 min to collect the supernatant, and the TAM cell culture supernatant was collected followed by centrifugation at 12,000 rpm for 15 min at 4 °C to remove cell debris, and immediately frozen at a − 80 °C freezer for later use. The levels of TNF, IL-1β, and IL-10 in the cell culture supernatant were measured using TNF (DTA00C), IL-1β (DLB50), and IL–10 (D1000B) ELISA kits, respectively as per the instructions of ELISA kits (R&D systems, Minneapolis, MN, USA). After the reaction was terminated, the absorbance (A) value of each well at 450 nm was measured using a totipotent microplate reader (Synergy2, BioTek, USA) within 10 min. With the concentration of standard substance as x-axis and A as y-axis, the regression equation of the standard curve was calculated, and the A value of the sample was substituted into the equation to calculate the target protein concentration in the sample.

### Dual luciferase reporter gene assay

The KDM5A target gene dual luciferase reporter gene vector and mutants at the binding site with ELK4 were constructed respectively, namely PGLO-KDM5A WT and PGLO-KDM5A MUT. The two reporter plasmids were co-transfected into the cells with sh-ELK4 and sh-NC, respectively. After 30 h of transfection, the cells were lysed, and centrifuged at 12,000 rpm for 1 min, and the supernatant was collected. The luciferase activity was detected by dual luciferase reporter assay system (E1910, Promega). In each cell sample, 100 μl firefly luciferase working solution was added to detect firely luciferase, and 100 μl Renilla luciferase working solution was added to detect Renilla luciferase. The ratios between firefly luciferase activity to Renilla luciferase activity were used as relative luciferase activities.

### Chromatin immunoprecipitation (ChIP)

ChIP kit (MilliporeCorp., Billerica, MA, USA) was used. TAMs were collected fixed with 1% formaldehyde at room temperature for 10 min. DNA and protein were immobilized and crosslinked. After crosslinking, they were randomly broken by ultrasonic treatment into 200–1000 base pairs. After centrifugation, the supernatant was divided into three tubes. Positive control antibody RNA polymerase II, negative control antibody rabbit anti IgG (ab172730, 1:100, Abcam) and rabbit anti-H3K4me3 (1:200, ab8580, Abcam) were added respectively. The endogenous DNA protein complex was precipitated by Agarose/Sepharose, the supernatant was aspirated after centrifugation, the nonspecific complex was washed, the de-crosslinking was carried out overnight at 65 °C, and the DNA fragment was recovered by phenol/chloroform extraction and purification. The promoter sequence of PJA2 gene and the DNA recovered from antibody bound chromatin fragment were detected by semi-qPCR.

### Co-immunoprecipitation (co-IP)

TAMs were cleaved on ice using IP lysate (Baimeiouxidi Biotechnology Co., Ltd., Wuxi, China) with protease inhibitor (MG-132). Next, 1 mg protein was extracted from each sample, and the volume was adjusted to the same using IP lysate, followed by addition of KSR1 monoclonal antibody for co-IP, and incubation in a silent mixer at 4 °C overnight. A total of 20 μl protein A + G beads were added in the morning of the next day and incubated for 2 h. After centrifugation, the supernatant was discarded carefully and 20 μl 2× loading buffer was added into each well. The samples were subjected to SDS-PAGE and analyzed by Western blot. The antibodies used were: anti IgG (1:100, ab172730, Abcam) and anti Ub (1:100, ab7780, Abcam).

### Protein stability test

To determine the stability of KSR1 protein, PJA2 was overexpressed in TAMs and incubated with 20 µg/ml CHX (protein synthesis inhibitor, purchased from Sigma-Aldrich Chemical Company) for a specified time. The cells were lysed with RIPA lysate (P0013B, Beyotime) and centrifuged at 12,000*g*/minute. After protein extraction, cells were lysed in RIPA buffer containing 0.1% sodium dodecyl sulfate for performing Western blot analysis. The expression level of KSR1 was quantified by ImageJ and normalized to GAPDH. The protein was extracted at 0, 1 and 2 h, and the expression of KSR1 was detected by Western blot.

### Cell counting kit-8 (CCK-8) assay

HGC-27 proliferation was statistically analyzed by CCK-8 method (CK04, Dojindo Laboratories, Kumamoto, Japan). The absorbance value of each well at 450 nm wavelength was measured by enzyme-linked immunosorbent assay. The value was directly proportional to the number of proliferating cells in the medium, and the cell growth curve was drawn.

### Transwell assay

Invasion and migration activity of the cells was analyzed using a 24-well Transwell chamber (8 μm aperture, Corning, USA) coated with/without Matrigel (BD Biosciences, Franklin Lakes, NJ, USA) [21]. The stained cells were manually counted under inverted light microscope (Carl Zeiss MicroImaging, Inc., Thornwood, NY, USA) in randomly selected five regions.

### Tumorigenesis in nude mice

Eighteen specific-pathogen-free grade female BALB/C nude mice (4–5 weeks, 18–22 g) were purchased from SLAC (Shanghai, China). They were raised in specific-pathogen-free environment for adaptive feeding for 7 days, with comfortable temperature, aseptic feed and drinking water, under alternate day and night cycles each for 12 h. TAMs stably transfected with sh-NC + oe-NC, sh-ELK4 + oe-NC and sh-ELK4 + oe-KSR1 were screened. The stable transfected TAMs (5 × 10^5^ cells/ml) and HGC-27 (1 × 10^6^ cells/ml) were inoculated into the left axillary skin of nude mice to establish the subcutaneous transplantation tumor model in nude mice. One week after inoculation, tumor growth was observed and data were recorded every 3 days. On the 26th day of culture, mice were killed by cervical dislocation. Tumor tissue was taken out, and tumor weight was weighed with a balance. The related mRNA expression in transplanted tumor tissue was detected by RT-qPCR.

### Statistical analysis

All the data in this study were analyzed using the SPSS 21.0 statistical software (IBM, Armonk, NY, USA). Each experiment was repeated three times. The measurement data were expressed by mean ± standard deviation. Paired *t* test was used to compare data between cancer tissues and adjacent tissues. Data between two groups were compared by independent sample *t* test, and those among multiple groups by one-way analysis of variance (ANOVA), followed by Tukey’s post hoc tests. Tumor volume at different time points were compared by repeated measures of ANOVA, followed by Bonferroni’s post hoc tests. For comparison of proliferation data at different time points, two-way ANOVA was used. *p* < 0.05 meant statistically significant difference.

## Results

### M2 polarization of macrophages promoted GC development

**Fig. 1:**
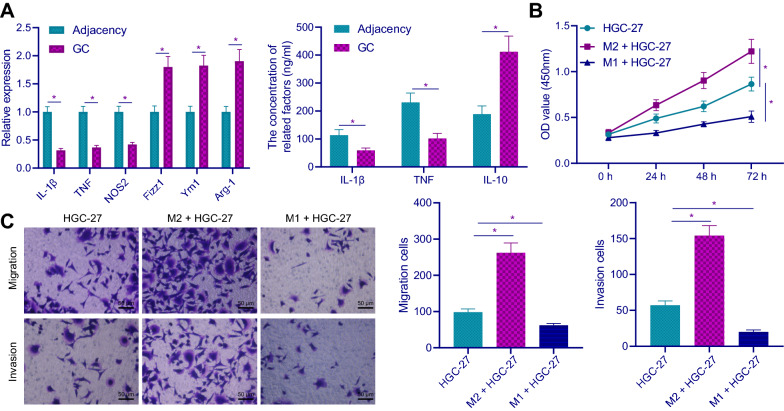
M2 polarization of macrophages promotes GC development. **A** The mRNA and protein expression levels of M1 and M2 macrophage markers in GC tissues and adjacent tissues of GC patients were detected by RT-qPCR and ELISA, respectively (n = 30). **B** The proliferation of HGC-27 cells was detected by CCK-8 assay after co-culture of M2 and M1 macrophages with HGC-27. **C** The migration and invasion of HGC-27 cells were detected by Transwell assay after co-culture of M2 and M1 macrophages and HGC-27 cells (× 200). *p < 0.05

To study the effect of macrophage polarization on GC cells, firstly, RT-qPCR was used to detect the M1 and M2 polarization of macrophages in GC tissues and adjacent tissues. The results showed that the mRNA expression levels of M1 markers: IL-1β, TNF and NOS2 in GC tissues were decreased, while the mRNA expression levels of M2 markers: Fizz1, Ym1 and Arg-1 were increased (Fig. 1A), indicating that macrophages mainly polarized to M2 type in GC tissues. ELISA results indicated that M1 markers: IL-1β and TNF were decreased and M2 marker: IL-10 was increased in GC tissues (Fig. 1A). Then, M2 macrophages and M1 macrophages were co-cultured with GC cell line HGC-27. Besides, after co-culture of M2 macrophages and HGC-27, the proliferative, migrative and invasive potentials of HGC-27 cells were enhanced. Additionally, the proliferative, migrative and invasive potentials of HGC-27 cells were inhibited after co-culture of M1 macrophages with HGC-27 cells (Fig. 1B, C). All in all, M2 polarization of macrophages promoted the proliferative, migrative and invasive potentials of HGC-27 cell.

### ELK4 was highly expressed in GC tissues and TAMs

**Fig. 2:**
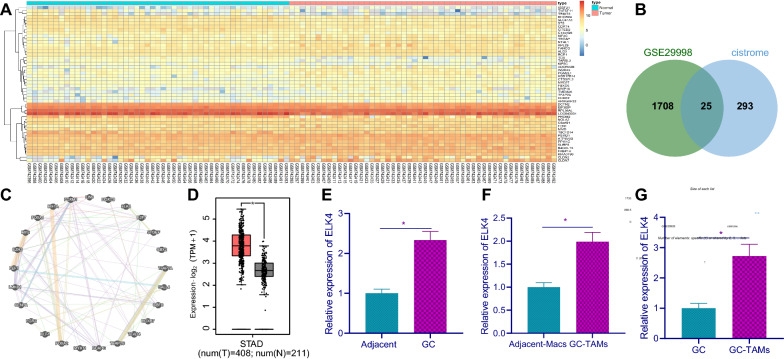
ELK4 is highly expressed in GC tissues and TAMs. **A** The expression heat map of some differentially expressed genes including hub genes in GC dataset GSE29998. The abscissa represents the sample number, the ordinate represents the gene name, and the left dendrogram represents the expression level clustering. Each small block in the graph represents the expression of a gene in a sample, and the histogram at the upper right is the color scale. **B** The overlap of significantly different gene in GSE29998 and transcription factors collected in cistrome. **C** Candidate transcription factor correlation analysis. Each small circle in the graph represents a gene, and the connection between genes indicates that there is correlation between genes. D, The expression of ELK4 gene in GC data collected by TCGA and GTEX. The abscissa represents sample type, ordinate represents expression value, red box diagram represents tumor samples, and the gray box diagram represents normal samples. (T: Tumor; N, Normal; *p < 0.01). E, RT-qPCR was used to detect the relative mRNA expression level of ELK4 in GC and adjacent tissues (n = 30). F, RT-qPCR was used to detect the mRNA expression of ELK4 in GC-TAMs and in Macs in adjacent tissues of GC patients. G, RT-qPCR was used to detect the mRNA expression of ELK4 in GC tissues and GC-TAMs. *p < 0.05

GC expression microarray GSE29998 was obtained from GEO database, and 1733 differentially expressed genes were obtained by differential analysis. The heat map of some of the differentially expressed genes is shown in Fig. 2A. Furthermore, the known transcription factor data (Fig. 2B) were obtained through cistrome database. Finally, 25 hub genes were obtained. These 25 hub genes are not only differentially expressed in GC, but also play a transcriptional regulatory role as transcription factors. Further correlation analysis of these 25 candidate transcription factors (Fig. 2C, Additional file 2: Table S2) showed that the three transcription factors scored the highest: ELK4, LIN9 and HOXC9. Among the three transcription factors, we found ELK4 showed the highest expression in the microarray GSE29998 (Table 1). In addition, compared with GC and normal samples in TCGA and GTEX databases, ELK4 was also highly expressed in GC tissues (Fig. 2D). We further verified through RT-qPCR that ELK4 mRNA expression in GC tissues was significantly higher than that in adjacent tissues (Fig. 2E). Next, TAMs and macrophages (Macs) were isolated from GC and adjacent tissues followed by determination of ELK4 mRNA expression, and the obtained results were consistent with those in tissues (Fig. 2F). In addition, we examined ELK4 expression in GC tissues and GC-TAMs and found that ELK4 expression was high in GC-TAMs (Fig. 2G). Moreover, the correlation between ELK4 mRNA expression with M1 and M2 markers is shown in Additional file 3: Table S3. The results showed that ELK4 was upregulated in GC tissues and TAMs and mainly expressed in GC-TAMs.

**Table 1:** Differential expression of candidate transcription factors in microarray

Symbol	logFC	Ave expr	p.value	Adj.p.val
ELK4	1.492757041	2.278704623	0.000941832	0.005681043
LIN9	1.266602945	4.600122208	3.63E−06	4.61E−05
HOXC9	1.067821739	4.403106717	0.002149055	0.01153422

*ELK4* ETS-like transcription factor 4, *HOXC9* homeobox C9

### ELK4 promoted M2 polarization of macrophages through transcriptional activation of KDM5A

**Fig. 3:**
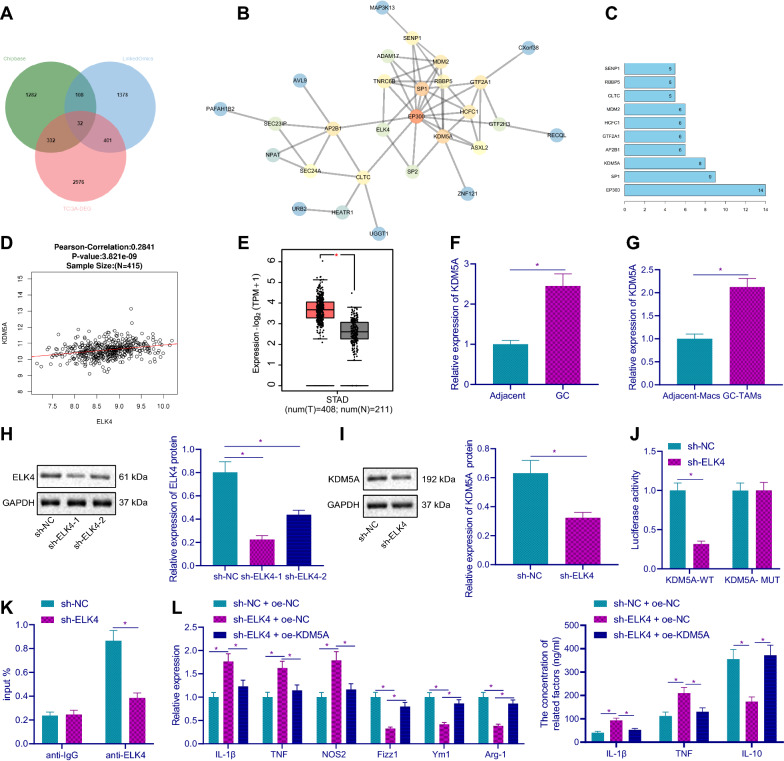
ELK4 promotes macrophage M2 polarization through transcriptional activation of KDM5A. **A** Prediction of ELK downstream target gene. The three circles in the Figure represent the prediction results of ChipBase database, LinkedOmics database and significantly different genes in TCGA and GTEX, and the middle part represents the intersection of the three groups of data. **B** The interaction analysis of ELK4 downstream target genes. Each small circle in the figure represents a gene, and the line between circles indicates the interaction between genes. A darker circle color indicates a higher number of interacting genes, a higher degree value and a higher core degree in the network graph. **C** The statistics of the core gene degree value in the gene interaction network graph. The abscissa represents the degree value, and the ordinate represents the gene name. **D** The correlation analysis results of ELK4 and KDM5A in TCGAGC data. The abscissa represents ELK4 expression, and ordinate represents KDM5A. Each small circle in the graph represents a sample, and the person correlation coefficient and correlation *p* value are shown above. **E** The expression of KDM5A in GC data collected by TCGA and GTEX (T: Tumor. N, Normal. *p < 0.01). **F** The mRNA expression level of KDM5A in GC tissues and adjacent tissues of GC patients (n = 30). **G** RT-qPCR was used to detect KDM5A mRNA expression in GC-TAMs and in Macs of adjacent tissues. **H** After silencing ELK4 in TAMs, the protein expression of ELK4 was detected by Western blot. **I** After ELK4 was silenced in TAMs, the protein expression of KDM5A was detected by Western blot. **J** After ELK4 was silenced in TAMs, the effect of ELK4 on KDM5A activity was detected by dual luciferase reporter gene assay. K, ELK4 was silenced in TAMs, ChIP assay was used to detect the enrichment of ELK4 on KDM5A promoter. **L** RT-qPCR and ELISA were used to detect the mRNA and protein expression of M1 and M2 macrophage markers in TAMs. *p < 0.05

To further understand the regulatory mechanism of ELK4 promoting M2 polarization of macrophages, the downstream regulatory genes of ELK4 were predicted through ChipBase database. Meanwhile, the genes with significant positive correlation with ELK4 in GC included in TCGA were searched through LinkedOmics database. The genes with significant differential expression in TCGA and GTExGC were obtained through GEPIA database, and the intersection of these three groups of data was taken (Fig. 3A). Finally, 32 hub genes were obtained. Then, we analyzed the gene interaction of these 32 candidate hub genes, constructed the gene interaction network graph, and counted the degree value of core genes in the network diagram (Fig. 3B, C). The results showed that KDM5A, SP1 and EP300 genes were in the core position in the network diagram. Among these three genes, KDM5A and ELK4 were positively correlated in GC (Fig. 3D), and in GC data collected by TCGA and GTEX, it was also overexpressed in GC tissues (Fig. 3E). We found that KDM5A mRNA expression was increased in GC tissues and TAMs (Fig. 3F, G). After silencing ELK4 in TAMs, we found decreased ELK4 protein expression by Western blot results (Fig. 3H), among which sh-ELK4-1 had the highest silencing efficiency, so it was selected for subsequent experiments. Further study on the regulatory mechanism of ELK4 on KDM5A showed that the protein expression of KDM5A was decreased after ELK4 was silenced (Fig. 3I). Luciferase assay demonstrated that compared with sh-NC group, in the co-transfection group with KDM5A-WT, the luciferase activity in sh-ELK4 group was decreased, while in the co-transfection group with KDM5A-MUT, no significant difference was found in luciferase activity between the two groups (Fig. 3J). ChIP experiment results showed that (Fig. 3K) after silencing ELK4, ELK4 enrichment on KDM5A promoter was reduced. The results showed that ELK4 could bind to KDM5A promoter and promote its transcription in TAMs.

Next, RT-qPCR and ELISA in TAMs showed (Fig. 3L) compared with sh-NC + oe-NC group, the mRNA expression levels of IL-1β, TNF and NOS2 and the protein expression of IL-1β and TNF in sh-ELK4 + oe-NC group were increased, while the mRNA expression levels of Fizz1, Ym1 and Arg-1 and the protein expression of IL-10 were decreased, indicating that silencing ELK4 could promote the M1 polarization of macrophages. Compared with sh-ELK4 + oe-NC group, the mRNA expression of IL-1β, TNF and NOS2 and the protein expression of IL-1β and TNF in sh-ELK4 + oe-KDM5A group was decreased, while the mRNA expression of Fizz1, Ym1 and Arg-1 and the protein expression of IL-10 was increased. These results suggested that ELK4 promoted M2 polarization of macrophages through transcriptional activation of KDM5A.

### KDM5A inhibited PJA2 expression by removing H3K4me3, thus promoting M2 polarization of macrophages

**Fig. 4:**
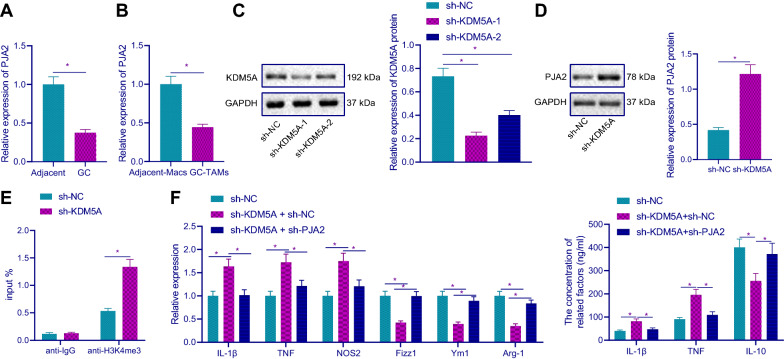
KDM5A inhibits PJA2 expression by removing H3K4me3 and promoted macrophage M2 polarization. **A** The mRNA expression of PJA2 was detected by RT-qPCR (n = 30). **B** The mRNA expression of PJA2 in GC-TAMs and Macs of adjacent tissues was detected by RT-qPCR. **C** KDM5A protein expression was detected by Western blot after KDM5A was silenced in TAMs. **D** After KDM5A was silenced in TAMs, Western blot was used to detect the protein expression of PJA2. **E** After KDM5A was silenced in TAMs, the enrichment of H3K4me3 on PJA2 promoter was detected by ChIP assay. **F** The mRNA and protein expression of M1 and M2 macrophage markers in TAMs was detected by RT-qPCR and ELISA, respectively. *p < 0.05

Studies have shown that PJA2 can promote M1 macrophage polarization, and the low expression of PJA2 is associated with the poor prognosis of GC [11, 12]. Through RT-qPCR, we found decreased PJA2 mRNA expression in GC tissues and TAMs (Fig. 4A, B). Western blot confirmed the silencing efficiency of KDM5A and sh-KDM5A-1 had the highest silencing efficiency was selected for subsequent experiments (Fig. 4C). Besides, after silencing KDM5A, PJA2 protein expression was increased (Fig. 4D). ChIP experiment results showed that (Fig. 4E) after silencing KDM5A, H3K4me3 was enriched in the promoter region of PJA2. The aforementioned results demonstrated that KDM5A could inhibit PJA2 expression by removing the enrichment of H3K4me3 in the promoter region of PJA2.

Next, RT-qPCR and ELISA in TAMs showed (Fig. 4F) compared with sh-NC group, the mRNA expression of IL-1β, TNF and NOS2 and the protein expression of IL-1β and TNF in sh-KDM5A + sh-NC group was increased, while the mRNA expression of Fizz1, Ym1 and Arg-1 and the protein expression of IL-10 was decreased; compared with sh-KDM5A + sh-NC group, the mRNA expression of IL-1β, TNF, and NOS2 and the protein expression of IL-1β and TNF in sh-KDM5A + sh-PJA2 group were lower, while the expression of Fizz1, Ym1 and Arg-1 and the protein expression of IL-10 increased than those in sh-KDM5A + sh-NC group. These results indicated that KDM5A decreased PJA2 expression by removing H3K4me3, thus inducing M2 polarization of macrophages.

### PJA2 decreased KSR1 expression by ubiquitination to inhibit M2 polarization of macrophages

**Fig. 5:**
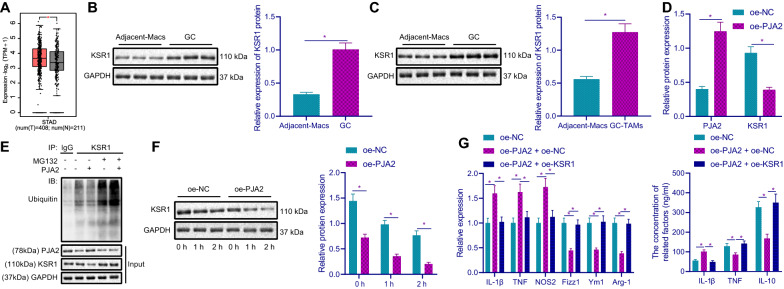
PJA2 decreases KSR1 by ubiquitination, thus suppressing macrophage M2 polarization. **A** The expression of KSR1 in GC data collected by TCGA and GTEX (T: Tumor. N: normal. *p < 0.05). **B** KSR1 protein expression level in GC tissues and adjacent tissues of GC patients was detected by Western blot (n = 30). **C** KSR1 protein expression levels in TAMs in GC tissues and in Macs of adjacent tissues was detected by Western blot. **D** After overexpression of PJA2 in TAMs, Western blot was used to detect the protein expression of KSR1. **E** After overexpression of PJA2 in TAMs, the binding of KSR1 to ubiquitin was detected by co-IP assay. **F** The stability of KSR1 protein was detected by CHX treatment after overexpression of PJA2 in TAMs. **G** The mRNA and protein expression levels of M1 and M2 macrophage markers in TAMs cells were detected by RT-qPCR and ELISA. *p < 0.05

The GC data in TCGA and GTEX showed that KSR1 was highly expressed in GC tissues (Fig. 5A). Western blot results showed that KSR1 protein expression was significantly increased in GC tissues and TAMs (Fig. 5B, C). Besides, after overexpression of PJA2, PJA2 protein expression was increased, while KSR1 protein expression was decreased (Fig. 5D). To further study the regulatory mechanism of PJA2 on KSR1, co-IP assay was used to detect the effect of overexpression of PJA2 on the ubiquitination level of KSR1. The results showed that (Fig. 5E) after the addition of MG132, the binding amount of KSR1 and ubiquitin increased upon overexpression of PJA2, that is, the ubiquitination level of KSR1 increased. After CHX treatment, the stability of KSR1 protein was detected. The results revealed that the protein stability of KSR1 was decreased after overexpression of PJA2. These findings supported that PJA2 was able to reduce the expression of KSR1 by ubiquitination in TAMs.

Next, we found from RT-qPCR and ELISA (Fig. 5G) that compared with oe-NC group, the mRNA expression of IL-1β, TNF and NOS2 and the protein expression of IL-1β and TNF in oe-PJA2 + oe-NC group was increased, while the mRNA expression of Fizz1, Ym1 and Arg-1 and the protein expression of IL-10 was decreased; compared with oe-PJA2 + oe-NC group, the expression of IL-1β, TNF and NOS2 and the protein expression of IL-1β and TNF in oe-PJA2 + oe-KSR1 group were lower, but the expression of Fizz1, Ym1 and Arg-1 and the protein expression of IL-10 increased than those in oe-PJA2 + oe-NC group.

These findings demonstrated that PJA2 reduced KSR1 expression through ubiquitination, thus inhibiting macrophage M2 polarization.

### ELK4 induced M2 polarization of macrophages by KSR1 to promote the proliferative, migrative and invasive abilities of GC cells

**Fig. 6:**
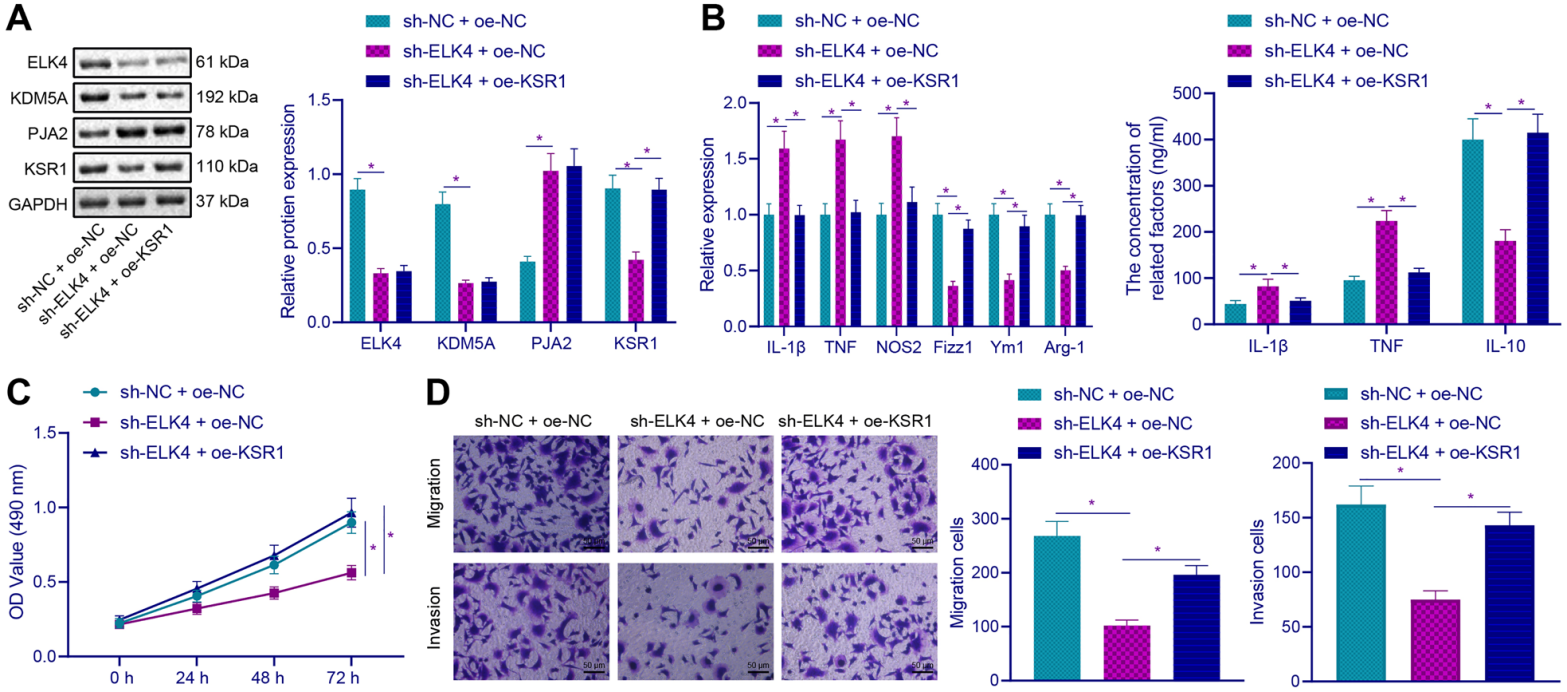
ELK4 promotes M2 polarization of macrophages by regulating KSR1 to promote the proliferation, migration and invasion of GC cells. **A** Western blot was used to detect the expression of ELK4, KDM5A, PJA2 and KSR1 in TAMs. **B** mRNA and protein expression of M1 and M2 macrophage markers in TAMs cells was detected by RT-qPCR and ELISA. **C** The proliferation of HGC-27 cells was detected by CCK-8 assay after TAMs were co-cultured with HGC-27. **D** After TAMs were co-cultured with HGC-27 cells, the migration and invasion of HGC-27 cells were detected by Transwell assay (× 200). *p < 0.05

Based on the above studies, we speculate that ELK4 may regulate macrophage M2 polarization through KDM5A-PJA2-KSR1 axis, thus affecting the biological function of GC cells. First, we observed (Fig. 6A, B) that compared with sh-NC + oe-NC group, the protein and mRNA expression levels of ELK4, KDM5A and KSR1 in sh-ELK4 + oe-NC group were significantly decreased, and the protein and mRNA expression of PJA2 was increased; compared with sh-ELK4 + oe-NC group, the protein and mRNA expression of ELK4, KDM5A and PJA2 in sh-ELK4 + oe-KSR1 group was not changed, but KSR1 protein and mRNA expression was increased. Besides, ELISA revealed that compared with sh-NC + oe-NC group, sh-ELK4 + oe-NC markers: IL-1β, TNF and NOS2 mRNA and IL-1β and TNF protein expression were increased, while M2 markers: Fizz1, Ym1, and Arg-1 mRNA and IL-10 protein expression were decreased; compared with sh-ELK4 + oe-NC group, sh-ELK4 + oe-KSR1 markers: IL-1β, TNF, and NOS2 mRNA and IL-1β and TNF protein expression were decreased, while M2 markers: Fizz1, Ym1 and Arg-1 mRNA and IL-10 protein expression were increased. After TAMs were co-cultured with HGC-27, CCK-8 and Transwell detection results showed (Fig. 6C, D) that compared with sh-NC + oe-NC group, the proliferative, migrative and invasive abilities of HGC-27 in sh-ELK4 + oe-NC group were decreased; compared with sh-ELK4 + oe-NC group, the proliferative, migrative and invasive abilities of HGC-27 in sh-ELK4 + oe-KSR1 group were increased. The results demonstrated that ELK4 could promote the proliferative, migrative and invasive potentials of GC cells by regulating KSR1.

### ELK4 promoted macrophage M2 polarization by regulating KSR1, thus promoting the growth of GC xenografts

**Fig. 7:**
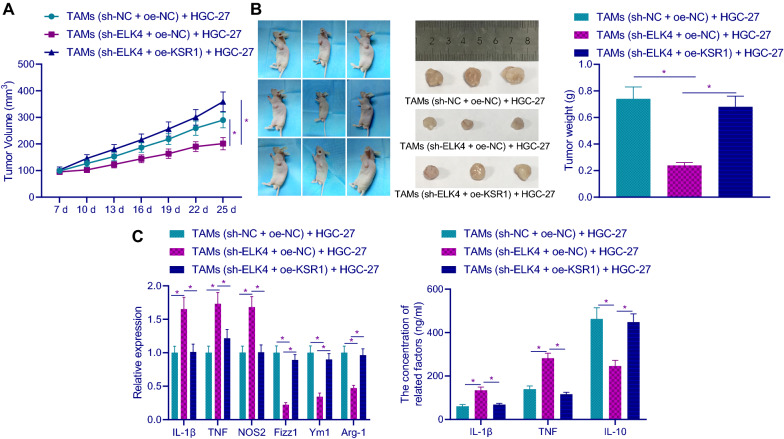
ELK4 promotes macrophage M2 polarization by regulating KSR1, thus promoting the growth of GC xenografts. **A** The tumor volume in mice in each group. **B** The tumor weight in mice in each group and the representative images. **C** The mRNA and protein expression of M1 and M2 macrophage markers was detected by RT-qPCR and ELISA. n = 6. *p < 0.05. Data among multiple groups by one-way ANOVA, followed by Tukey’s post hoc tests

In order to further study the effect of ELK4 on GC xenografts by regulating KSR1-mediated M2 polarization of macrophages, we conducted tumorigenesis experiment in nude mice. TAMs after different treatment and HGC-27 cells were inoculated subcutaneously in nude mice. It was found (Fig. 7A, B) that compared with sh-NC + oe-NC group, the volume and weight of transplanted tumor in sh-ELK4 + oe-NC group were reduced; compared with sh-ELK4 + oe-NC group, the tumor volume and weight were increased in sh-ELK4 + oe-KSR1 group. Then, compared with sh-NC + oe-NC group, the mRNA expression of IL-1β, TNF and NOS2 and the protein expression of IL-1β and TNF in sh-ELK4 + oe-NC group was increased, while the mRNA expression of Fizz1, Ym1 and Arg-1 mRNA and the protein expression of IL-10 was decreased; compared with sh-ELK4 + oe-NC group, the mRNA expression of IL-1β, TNF and NOS2 and the protein expression of IL-1β and TNF were higher while the mRNA expression of Fizz1, Ym1 and Arg-1 and the protein expression of IL-10 increased in sh-ELK4 + oe-KSR1 group (Fig. 7C).

These results concluded that ELK4 promoted macrophage M2 polarization by regulating KSR1, thus promoting the growth of GC xenografts.

## Discussion

Gastric cancer is considered to be a frequently occurring malignancy presenting a poor prognosis [22]. In the current study, we explored the regulatory mechanism of ELK4 in GC and found that ELK4 promoted the development of GC by regulating the PJA2/KDM5A/KSR1 axis.

Initially, we found that M2 polarization of macrophages promoted the development of GC. It is known that TAMs are representative of a main subpopulation of tumor infiltrating immune cells [23]. It was found that TAMs of the M2 phenotype are responsible for the progression of peritoneal dissemination in GC [24]. Moreover, M2 macrophage polarization induced by GC-derived mesenchymal stromal cells was revealed to enhance metastasis and epithelial to mesenchymal transition in GC [25]. Furthermore, we demonstrated that ELK4 was highly expressed in GC and TAMs and could promote M2 polarization of macrophages. ELK4 is involved in the development of prostate cancer by regulating the chimeric fusion SLC45A3-ELK4 transcript [26]. Additionally, a previous study found that ELK4 is correlated with the survival of glioblastoma patients and thus serves as a potential prognostic marker [27]. Intriguingly, it was unfolded that ELK4 could play an important role in regulating cellular homeostasis as well as stress responses in macrophages, thereby accelerating acute responses to external infection [7].

Mechanistically, we demonstrated that ELK4 inhibited PJA2 expression through transcriptional activation of KDM5A, which promoted M2 polarization of macrophages. Of note, the interaction between ELK4 and KDM5A and that between ELK4 and PJA2 have been rarely reported. Strikingly, mounting evidence has highlighted the role of KDM5A and PJA2 in GC. For instance, Zeng et al*.* discovered overexpression of KDM5A in GC and its inhibition triggers senescence of cancer cells [28]. Moreover, KDM5A could induce the promotion of gastric tumorigenesis through modulation of VEGF expression transactivation together with elevated angiogenesis, which thus affected the development and progression of human GC [29]. Notably, downregulated PJA2 shared correlation with worse overall survival of patients with GC and PJA2 was thus suggested as a potential plasma circRNA biomarker for this cancer type [12].

Another important finding obtained in the study was that PJA2 decreased KSR1 by ubiquitination and thus inhibited M2 polarization of macrophages. Recent evidence pronounced that PJA2 could induce ubiquitylation of MFHAS1, thereby promoting M1 macrophage polarization through activation of JNK and p38 pathways [30]. Another study revealed that PJA2 contributed to MFHAS1 ubiquitylation, positively regulating TLR2-mediated JNK/p38 pathway, which stimulated M1 macrophage polarization as well as M2 to M1 macrophage transformation in sepsis [11]. Importantly, the interaction between PJA2 and KSR1 has been previously reported. It was found that PJA2 is able to ubiquitylate KSR1 to regulate the growth of cancer cells [13]. Of note, upregulation of KSR1 was found in SGC-7901/CDDP cell resistant to human GC multidrug resistant and activated KSR1-mediated ERK1/2 pathway resulted in tumorigenesis in human GC [31].

## Conclusion

**Fig. 8:**
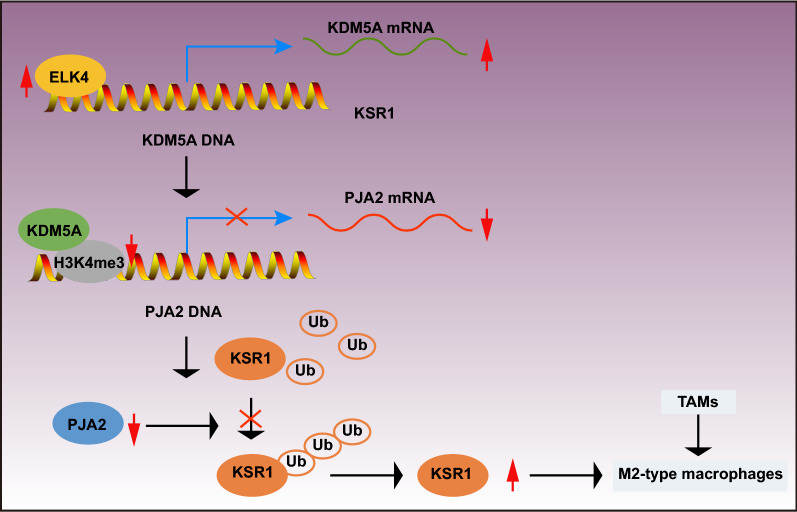
The molecular mechanism plot for the role of ELK4 in GC by regulating KDM5A/PJA2/KSR1 axis. ELK4 may inhibit PJA2 expression through transcriptional activation of KDM5A, which reduces the ubiquitination of KSR1, promoting M2 polarization of macrophages and thus facilitating the development of GC

To sum up, our results demonstrate that ELK4 may inhibit PJA2 expression through transcriptional activation of KDM5A, thereby reducing the ubiquitination of KSR1, which promotes M2 polarization of macrophages and thus promotes the development of GC (Fig. 8). This finding may provide a novel direction for treatment of GC. Nevertheless, further study is required to validate the mechanism and to explore the clinical application.

## Supplementary Information

**Additional file 1: Table S1.** Primer sequences for RT-qPCR.

**Additional file 2: Table S2.** Correlation analysis of these 25 candidate transcription factors.

**Additional file 3: Table S3.** The correlation between ELK4 mRNA expression with M1 and M2 markers.

## Data Availability

The datasets generated and/or analysed during the current study are available from the corresponding author on reasonable request.

## Abbreviations

GC: Gastric cancer
TAMs: Tumor-associated macrophages
ELK4: ETS-like transcription factor 4
PJA2: Praja2
KSR1: Kinase suppressor of ras 1
ERK: Extracellular signal-regulated kinase
RT-qPCR: Reverse transcription-quantitative polymerase chain reaction
GAPDH: Glyceraldehyde-3-phosphate dehydrogenase
RIPA: Radioimmunoprecipitation assay
ChIP: Chromatin immunoprecipitation
co-IP: Co-immunoprecipitation
CCK-8: Cell counting kit-8
