# Supplementary material for: ELK4 promotes the development of gastric cancer by inducing M2 polarization of macrophages through regulation of the KDM5A-PJA2-KSR1 axis
Source: J Transl Med. 2021 Aug 9;19:342. doi: 10.1186/s12967-021-02915-1 (PMC8353876; doi:10.1186/s12967-021-02915-1)
Supplement: Supplementary file 1 — Additional file 1: Table S1. Primer sequences for RT-qPCR. [file 12967_2021_2915_MOESM1_ESM.docx]

**Table S1** Primer sequences for RT-qPCR

| Gene | Sequences |
| --- | --- |
| ELK4 | F: 5’-GGGTTAGAACTGGCACCCAC-3’ |
|  | R: 5’-GCTGGACTTAGGGGAGCAAC-3’ |
| KDM5A | F: 5’-CCGTCTTTGAGCCGAGTTG-3’ |
|  | R: 5’-GGACTCTTGGAGTGAAACGAAA-3’ |
| PJA2 | F: 5’-CATTTCGGAATCTTCTGCGGC-3’ |
|  | R: 5’-CATCACCAGCCCGACCTAAG-3’ |
| IL-1β | F: 5’-CCCTAAACAGATGAAGTGCTCC-3’ |
|  | R: 5’-ATCTTCCTCAGCTTGTCCATG-3’ |
| TNF | F: 5’-CACAGTGAAGTGCTGGCAAC-3’ |
|  | R: 5’-AGGAAGGCCTAAGGTCCACT-3’ |
| NOS2 | F: 5’-GCAGAGATTGGAGGCCTTGTG-3’ |
|  | R: 5’-GGGTTGTTGCTGAACTTCCAGTC-3’ |
| Fizz1 | F: 5’-AGCTCTCGTGTGCTAGTGTC-3’ |
|  | R: 5’-TGAACATCCCACGAACCACA-3’ |
| Ym1 | F: 5’-CAGGTCTGGCAATTCTTCTGAA-3’ |
|  | R: 5’-GTCTTGCTCATGTGTGTAAGTGA-3’ |
| Arg-1 | F: 5’-ACTTAAAGAACAAGAGTGTGATGTG-3’ |
|  | R: 5’-CATGGCCAGAGATGCTTCCA-3’ |
| GAPDH | F: 5’-ATGACCACAGTCCATGCCATC-3’ |
|  | R: 5’-GAGCTTCCCGTTCAGCTCTG-3’ |

Note: ELK4, ETS-like transcription factor 4; KDM5A, lysine-specific demethylase 5A; PJA2, Praja2; IL-1β, interleukin 1; TNF, tumor necrosis factor; NOS2, inducible nitric oxide synthase; Arf-1, arginase 1; GAPDH, glyceraldehyde-3-phosphate dehydrogenase; RT-qPCR, reverse transcription-quantitative polymerase chain reaction
