# Supplementary material for: ELK4 promotes the development of gastric cancer by inducing M2 polarization of macrophages through regulation of the KDM5A-PJA2-KSR1 axis
Source: J Transl Med. 2021 Aug 9;19:342. doi: 10.1186/s12967-021-02915-1 (PMC8353876; doi:10.1186/s12967-021-02915-1)
Supplement: Supplementary file 3 — Additional file 3: Table S3. The correlation between ELK4 mRNA expression with M1 and M2 markers. [file 12967_2021_2915_MOESM3_ESM.docx]

**Table S3** The correlation between ELK4 mRNA expression with M1 and M2 markers.

| ELK4 | IL-1β | TNM | NOS2 | Fizz1 | Ym1 | Arg-1 |
| --- | --- | --- | --- | --- | --- | --- |
| r | -0.5371 | -0.7072 | -0.6396 | 0.6608 | 0.5952 | 0.4531 |
| P | 0.0022 | <0.0001 | <0.0001 | <0.0001 | 0.0005 | 0.0119 |
